# Supplementary material for: Synthesis and Structural Insight into poly(dimethylsiloxane)-b-poly(2-vinylpyridine) Copolymers
Source: Polymers (Basel). 2023 Oct 25;15(21):4227. doi: 10.3390/polym15214227 (PMC10648597; doi:10.3390/polym15214227)
Supplement: Supplementary file 1 [file polymers-15-04227-s001.zip › polymers-2675346-supplementary.pdf]

Synthesis and Structural Insight into Poly(dimethylsiloxane)-*b*-Poly(2-vinylpyridine) Copolymers

Gkreti-Maria Manesi[1], Ioannis Moutsios[1,2], Dimitrios Moschovas[1], Georgios Papadopoulos[1], Christos Ntaras[1], Martin Rosenthal[3], Loic Vidal[2], Georgiy G. Ageev[4], Dimitri A. Ivanov[2,4,5,6] and Apostolos Avgeropoulos*[1,5]

[1] Department of Materials Science & Engineering, University of Ioannina, University Campus-Dourouti, 45110 Ioannina, Greece; gretimanesi@uoi.gr (G.-M.M.); imoutsios@uoi.gr (I.M.); dmoschov@uoi.gr (D.M.); gpap414@gmail.com (G.P.); ntaras@megaplast.gr (C.N.)

[2] Institut de Sciences des Matériaux de Mulhouse – IS2M, CNRS UMR7361, 15 Jean Starcky, Mulhouse 68057, France; loic.vidal@uha.fr (L.V.); dimitri.ivanov@uha.fr (D.A.I.)

[3] Department of Chemistry, KU Leuven, Celestijnenlaan 200F, Box 2404, B-3001 Leuven, Belgium; martin.rosenthal@esrf.fr (M.R.)

[4] Sirius University of Science and Technology, 1 Olympic Ave, 354340, Sochi, Russia; ageev.gg@talantiuspeh.ru (G.G.A)

[5] Faculty of Chemistry, Lomonosov Moscow State University (MSU), GSP-1, 1-3 Leninskiye Gory, 119991 Moscow, Russia;

[6] Federal Research Center of Problems of Chemical Physics and Medicinal Chemistry RAS, Russian Academy of Sciences, Chernogolovka, 142432 Moscow, Russia;

*Correspondence: aavger@uoi.gr (A.A.)

Supplementary Materials

The following data are given in the Supplementary Materials:

A). SEC Chromatograms of Living Homopolymers and Final Materials

B). Calculation of Mass Fractions

C). IR Spectra of Pristine and Modified Copolymers

A). SEC Chromatograms of Living Homopolymers and Final Materials

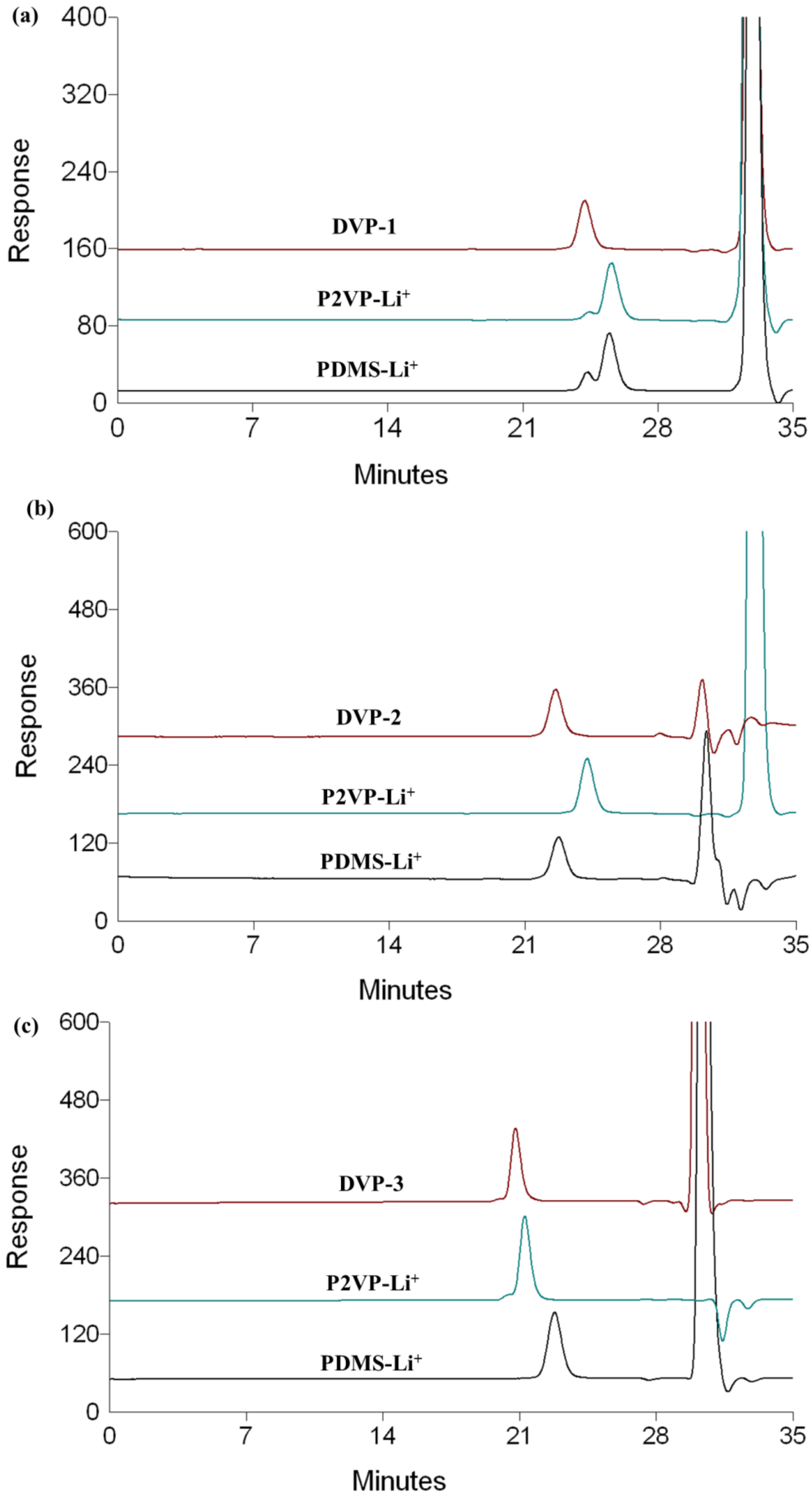

Figure S1: SEC chromatograms corresponding to: a) living PDMS-Li^+ homopolymer precursor (black) living P2VP-Li^+ homopolymer precursor (blue) and final diblock copolymer of the PDMS-*b*-P2VP type or DVP-1 (red), b) living PDMS-Li^+ homopolymer precursor (black) living P2VP-Li^+ homopolymer precursor (blue) and final diblock copolymer of the PDMS-*b*-P2VP type or DVP-2 (red) and c) living PDMS-Li^+ homopolymer precursor (black) living P2VP-Li^+ homopolymer precursor (blue) and final diblock copolymer of the PDMS-*b*-P2VP type or DVP-3 (red).

B). Calculation of Mass Fractions

The estimation of either PDMS or P2VP mass fractions through the integration values as directly calculated by the charectistic chemical shifts of the contributing protons (methyl groups at δ: 0.1-0.5 ppm for the PDMS block and proton of the aromatic ring at δ: 8.2 ppm for the P2VP segment) can be carried out using the following equations:

$$f_{PDMS} = \frac{integration\ value_{0.1-0.5}}{contributing\ protons\ (or\ 6)} x\ monomeric\ unit\ molecular\ weight\ (or\ 74\ g/mol),$$

$$f_{P2VP} = \frac{integration\ value_{8.20}}{contributing\ protons\ (or\ 1)} x\ monomeric\ unit\ molecular\ weight\ (or\ 105\ g/mol).$$

More specifically for DVP-3 the integration values of the contributing protons are 1 and 1.75 for the P2VP and PDMS respectively. As the result the above mentioned equations are transformed as follows:

$$f_{PDMS} = \frac{1.75}{6}\ x\ 74 = 21.5$$

$$f_{P2VP} = \frac{1.00}{1}\ x\ 105 = 105.0$$

It is already established that in a diblock copolymer the total mass fraction equals to one as indicated in the following equation: $f_{PDMS} + f_{P2VP} = 1$.

Therefore to calculate the mass fraction of PDMS segment the following calculations should be conducted: $f_{PDMS}=f_{PDMS}/f_{total}$ or $f_{PDMS}=21.5/(21.5+105) = 0.16$

while for the P2VP segment calculations are: $f_{P2VP}=f_{P2VP}/f_{total}$ or $f_{P2VP}=105/(21.5+105) = 0.84$.

As a result f_{PDMS}=16 % wt and f_{PDMS}=84 % wt.

A coherent process is applied in the remaining copolymers in order to calculate the mass fractions through ^{1}H NMR.

C). IR Spectra of Pristine and Modified Copolymers

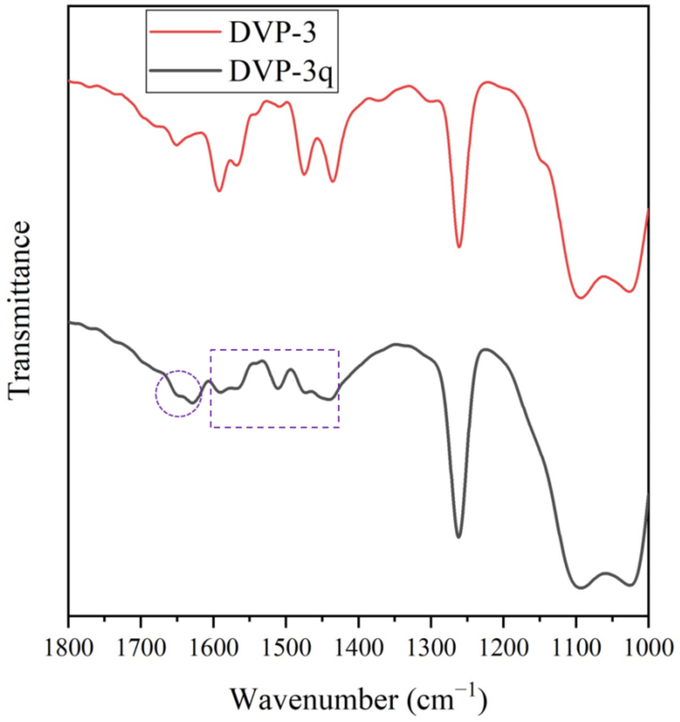

Figure S2: Magnified IR spectra corresponding to the DVP-3 and DVP-3q samples corresponding to the region between 1800 to 1000 cm^{-1}.
